# Simultaneous non-contrast assessment of cardiac microstructure and perfusion in vivo in the human heart

**DOI:** 10.1016/j.jocmr.2024.101129

**Published:** 2024-11-30

**Authors:** Camila Munoz, Eunji Lim, Pedro F. Ferreira, Dudley J. Pennell, Sonia Nielles-Vallespin, Andrew D. Scott

**Affiliations:** a National Heart and Lung Institute, Imperial College London, London, UK; b Royal Brompton and Harefield Hospitals, Guy’s and St Thomas’ NHS Foundation Trust, London, UK

**Keywords:** Diffusion tensor CMR, Intravoxel incoherent motion, Cardiac microstructure, Myocardial perfusion

## Abstract

**Background:**

Intravoxel incoherent motion (IVIM) imaging can provide information on cardiac microstructure and microvascular perfusion from a single examination. However, the spin echo-based approaches typically used for cardiac IVIM suffer from low sensitivity to changes in perfusion. The aim of this work was to develop a stimulated-echo (STEAM)-based method for IVIM and diffusion tensor cardiovascular magnetic resonance to simultaneously provide biomarkers of microstructure and perfusion in vivo in the human heart.

**Methods:**

Here we introduce a novel STEAM-IVIM sequence incorporating phase cycling to obtain true non-diffusion weighted images (b = 0 s/mm^2^). STEAM-IVIM imaging was performed at 20 b-values (0 to 1000 s/mm^2^) to enable accurate estimation of the IVIM parameters, and with six diffusion encoding directions to enable reconstruction of the diffusion tensor. 20 healthy subjects (8 female, median age 31 years) were imaged on a clinical 3T system with STEAM-IVIM. A simulation study was performed to investigate the optimal fitting algorithms for the IVIM parameters, which was subsequently used to create pixel-wise IVIM parameter maps for the in vivo acquisitions.

**Results:**

Good image quality across the myocardium was obtained for all b-values. Mean(±SD) IVIM parameter estimates were: diffusivity *D* = 0.83 ± 0.07 × 10-3 mm^2^/s, perfusion coefficient D* = 19.08 ± 6.48 × 10-3 mm^2^/s, perfusion fraction *f* = 19.72 ± 4.11%, and mean diffusion tensor parameters were: mean diffusivity = 0.88 ± 0.06 × 10-3 mm^2^/s, fractional anisotropy = 0.45 ± 0.04, absolute E2 angle = 55.29 ± 6.38º, helix angle gradient = -0.68 ± 0.18º/%.

**Conclusion:**

Phase-cycled STEAM-IVIM enables fitting of cardiac diffusion tensor and perfusion parameters in healthy subjects and shows promise for the simultaneous detection of microstructural aberration and perfusion abnormalities in the presence of cardiac disease without the need for exogenous contrast agents.

## Graphical abstract

**Figure:**
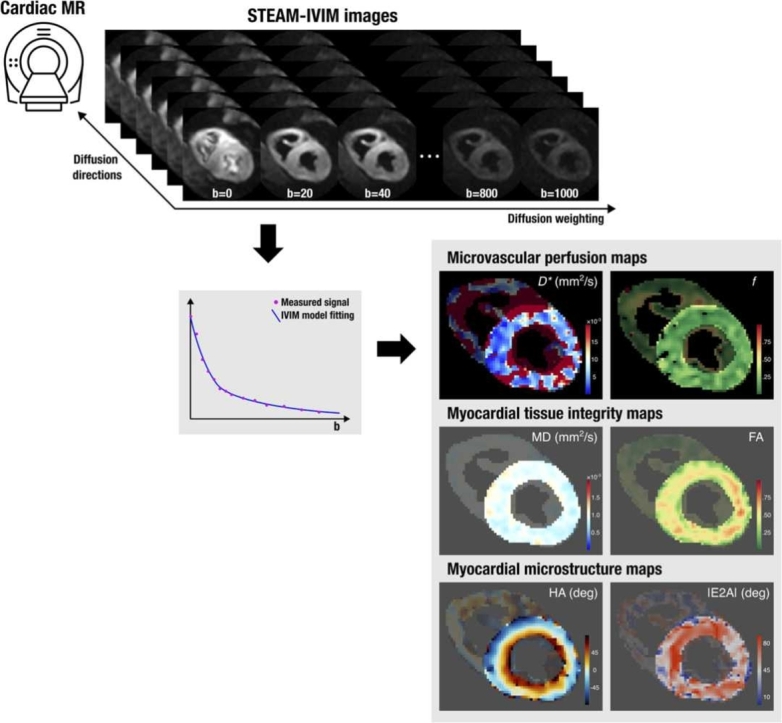
STEAM-IVIM method for the non-contrast assessment of myocardial microstructure and perfusion. Novel STEAM-IVIM sequence for the acquisition of diffusion-weighted images with diffusion weightings from 0 to 1000 s/mm^2^ and multiple diffusion encoding directions in vivo. Using an IVIM model fitting, maps reflecting microvascular perfusion can be obtained (D*: perfusion coefficient, *f*: perfusion fraction). Diffusion tensor (DT) analysis from the same data enables depiction of myocardial tissue integrity through mean diffusivity (MD) and fractional anisotropy (FA) maps, and depiction of myocardial microstructure, through maps of helix angle (HA) and absolute secondary eigenvector angle (E2A) reflecting cardiomyocyte orientation. *STEAM* stimulated echo acquisition mode, *IVIM* intravoxel incoherent motion

## 1 Introduction

Quantitative measures of myocardial perfusion obtained using cardiovascular magnetic resonance (CMR) have emerged as prognostically important biomarkers in ischemic [1] and non-ischemic cardiomyopathy [2]. To evaluate changes in microvascular blood flow within the myocardium, CMR perfusion imaging techniques measure changes in image contrast during the first pass of a gadolinium-based contrast agent bolus. This approach is routinely used in clinical CMR scans and can successfully measure changes in blood flow arising from exercise, disease, and pharmacological intervention. However, some gadolinium-based contrast agents are contraindicated in patients with impaired renal function and there are concerns regarding tissue deposition of gadolinium with repeated administration of the contrast agent [3], making contrast agent–free alternatives an important research target.

Non-contrast approaches proposed in the literature for the assessment of myocardial perfusion by CMR include arterial spin labeling and blood oxygen level-dependent (BOLD)–based methods 4, 5, 6. Arterial spin labeling uses magnetically labeled blood to assess myocardial perfusion, and although promising, it suffers from poor signal-to-noise ratio (SNR), which has hindered its clinical application. Myocardial BOLD imaging, on the other hand, is based on detecting variations in image contrast due to changes presence of oxyhemoglobin and deoxyhemoglobin in the blood. While these changes depend on blood flow in the myocardium, they are also affected by oxygen extraction fraction, and therefore this approach does not provide absolute measurements of myocardial perfusion. Furthermore, BOLD measures subtle differences in blood oxygenation levels, resulting in limited contrast-to-noise ratio between healthy and diseased areas.

An alternative approach to directly assess microvascular perfusion is based on the use of diffusion CMR imaging. Diffusion tensor CMR (DTCMR) is showing promise as a unique technique for the non-invasive contrast agent–free characterization of the myocardial microstructure in vivo in health and disease 7, 8, 9, 10. To probe the microstructure, diffusion tensor techniques measure the MR signal attenuation due to the incoherent thermal movement of water molecules in the presence of diffusion-encoding gradients. The movement of water molecules in the capillary network due to microvascular perfusion also has a high degree of incoherence and therefore contributes to this signal decrease and appears as a confounding factor on conventional diffusion imaging models, predominantly at low diffusion weightings (b-values) [11]. Alternatively, this phenomenon can be exploited to simultaneously obtain information about the myocardial microstructure and microvascular perfusion from a single scan and without the need for exogenous contrast agents. This framework, known as intravoxel incoherent motion (IVIM) imaging, models microvascular perfusion as a pseudo-diffusion process due to the random-like orientation of the vessels in the capillary network. Signal from both diffusing and perfusing spins can be then separately described by mono-exponential decay functions, so that the overall signal when acquired with a given diffusion-encoding strength *b*, *S*(*b*), is described by a bi-exponential decay as shown in Eq. (1).

(1) $$S(b)/S_{0}=f\exp(-bD^{*})+(1-f)\exp(-\mathrm{bD})$$

where *S*_0_ is a non-diffusion‐weighted signal, *f* is the flowing blood, *D* corresponds to the apparent diffusion coefficient, and D* is the perfusion coefficient (or pseudo-diffusion coefficient) [11].

The IVIM framework has been widely used in liver imaging, oncological, and neurological applications 12, 13, 14, but its application to cardiac imaging remains challenging. The deformation of the heart induced by the cardiac and respiratory cycles results in additional attenuation of the diffusion signal that can produce erroneous and indeed physically unrealistic estimations of the diffusion parameters [15]. To minimize the effect of breathing motion, conventional DTCMR studies typically use repeated breath-holding or respiratory navigator gating, while electrocardiogram (ECG) triggering and motion-robust diffusion encoding schemes have been used to minimize the effect of cardiac deformation. For cardiac IVIM in human subjects, studies have used ECG triggering to acquire data either during the end-systolic [16] or end-diastolic 17, 18 quiescent period of the cardiac cycle, in combination with repeated breath-holding [17], diaphragmatic navigator–based gating or slice tracking [18], or retrospective image–based motion correction [16]. In addition to ECG triggering, DTCMR acquisitions require motion-robust diffusion encoding schemes to avoid erroneous diffusivity measures 15, 19. Stimulated echo acquisition mode (STEAM) sequences [20] split diffusion encoding over two cardiac cycles, with short encoding gradients at identical points in successive cardiac cycles. Alternatively, motion–compensated diffusion encoding gradient waveforms can be used with spin echo (SE) sequences, where higher performance gradient systems are available and imaging in a systolic phase is suitable 21, 22, 23.

Recent studies have shown that in the heart, the choice of motion compensation or motion minimization strategies affects the sensitivity of the diffusion sequences to changes in microvascular perfusion. Indeed, Spinner et al. [24] and Alemany et al. [25] have shown in both simulation studies and controlled ex-vivo preclinical experiments that STEAM-based IVIM (STEAM-IVIM) has a higher sensitivity to changes in perfusion than SE-based IVIM. However, acquiring IVIM data using conventional STEAM DTCMR techniques remains challenging and has therefore never been performed in humans.

In conventional STEAM, spoiler gradients are used to remove residual stimulated anti-echo and free-induction decay components of the measured signal at low b-values [26]. However, these spoiler gradients contribute to the diffusion and perfusion weighting, preventing the use of STEAM for the acquisition of data with true b = 0 s/mm^2^. Indeed, the minimum achievable effective b-value when spoiler gradients are used is of the order of 20 s/mm^2^ for a heart rate of 60 beats per minute and a spatial resolution of 2.8 mm. If these spoiler gradients are removed the combined echoes prevent the true b = 0 s/mm^2^ stimulated echo being measured in isolation, and additional artifacts arise from the subcutaneous and epicardial fat in the free-induction decay component. Previous studies have omitted the acquisition of b = 0 images for STEAM-IVIM in perfused ex-vivo experiments [24]. Indeed, when IVIM is acquired with multiple data points, a b = 0 image can be estimated from the rest of the data, at the cost of increasing the number of unknown variables to be estimated and therefore an increase in the instability of the fitting and sensitivity to noise [11]. Due to these technical challenges, STEAM-IVIM has so far only been applied in small preclinical studies 24, 27. In this study, we further the capabilities of STEAM to enable for the first time the application of the STEAM-IVIM framework to the human heart in vivo. We used a phase-cycling approach to enable true b = 0 s/mm^2^ STEAM imaging and performed a simulation study to optimize the IVIM fitting algorithm. This enabled reliable fitting of the IVIM parameters in addition to the DTCMR measures of cardiac microstructure available from STEAM.

## 2 Methods

Twenty-three subjects (10 female, median age 33, range 20–59) were recruited for this study with informed consent according to ethical approval. The study protocol was approved by the London – Queen Square REC (19/LO/1014). Images were acquired using a 3T scanner (MAGNETOM Vida, Siemens Healthcare, Erlangen, Germany) with anterior (18 elements) and posterior (8–12 elements) radiofrequency (RF) coils. Balanced steady-state free-precession cine images were used to identify a mid-ventricular short-axis plane and the peak-systolic trigger delay for STEAM-IVIM imaging.

### 2.1 STEAM-IVIM acquisition

**Fig. 1:**
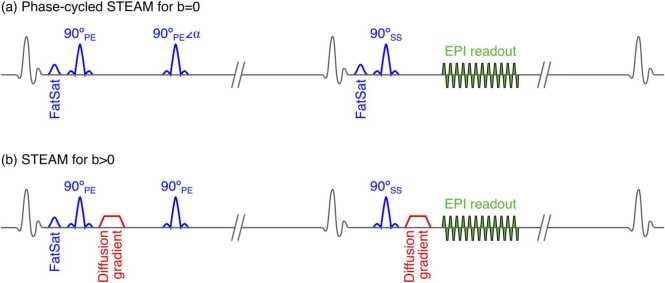
Schematic of the proposed STEAM-IVIM sequence. To reduce EPI duration, a zonal excitation was used (*PE* phase-encoding direction, *SS* slice selection direction). (a) For b = 0 s/mm^2^ images, a phase cycling of the second RF pulse of the sequence was used, so that in three consecutive acquisitions α = 0º, 120º, and 240º. A fat saturation pulse (FatSat) was included before the third RF pulse to further suppress the fat signal arising from the free-induction decay produced by the third RF pulse. (b) For b > 0 s/mm^2^ images, no phase cycling or secondary fat suppression was used. *STEAM* stimulated echo acquisition mode, *IVIM* intravoxel incoherent motion, *EPI* echoplanar imaging, *RF* radiofrequency

**Fig. 2:**
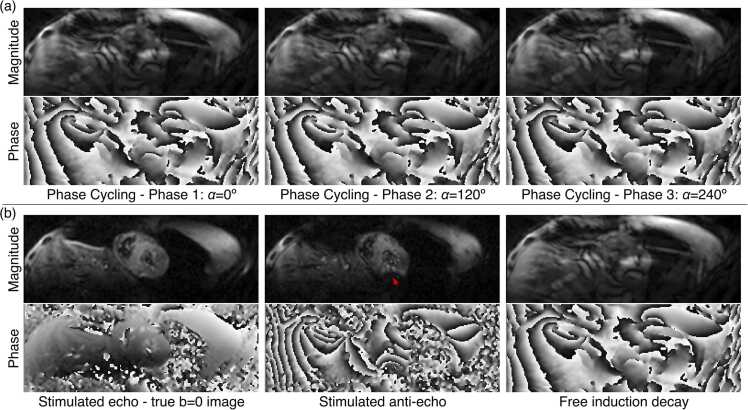
Example b = 0 STEAM-IVIM images. Example STEAM-IVIM phase-cycled b = 0 s/mm^2^ images, showing magnitude and phase images. (a) Input phase-cycled images with alpha = 0º, 120º, and 240º, where the desired stimulated echo is mixed with the stimulated anti-echo and high-intensity free-induction decay. (b) Output decoupled images. Good quality true b = 0 s/mm^2^ STEAM-IVIM images can be obtained (left column), with a homogeneous signal in the myocardium and corresponding smooth phase image, while T2* decay effects can be observed in the stimulated anti-echo image (middle column, red arrow). Wrap artifacts can be observed in the free-induction decay image (right column), which does not experience the reduced field of view in the phase encode direction. *STEAM* stimulated echo acquisition mode, *IVIM* intravoxel incoherent motion

A STEAM single-shot echoplanar (echoplanar imaging [EPI]) DTCMR sequence was implemented as described in [7]. To acquire true b = 0 s/mm^2^ STEAM images with no spoiler gradients, a three-point phase-cycling scheme was applied to the second RF 90° pulse of the STEAM sequence (Fig. 1) [28]. By exploiting phase differences induced by this scheme, the stimulated echo could be isolated (Fig. 2) 27, 29. In brief, in absence of diffusion encoding gradients, and when the phase of the second RF pulse is θ, the transverse magnetization available for imaging can be expressed as

(2) $$M_{\mathrm{xy}}(\theta )=\mathrm{STE}e^{i\theta }+\mathrm{STAE}e^{-i\theta }+\mathrm{FID}$$

Where STE is the desired stimulated echo, STAE is the stimulated anti-echo, and FID is the free induction decay signal. The three-point cycling approach acquires three consecutive datasets with θ= 0, 120º, or 240°, resulting in transverse magnetization as follows:

$$M_{\mathrm{xy}}\left(\theta =0\right)=\mathrm{STE}+\mathrm{STAE}+\mathrm{FID}$$

$$M_{\mathrm{xy}}\left(\theta =\frac{2\pi }{3}\right)=\mathrm{STE}e^{\frac{i2\pi }{3}}+\mathrm{STAE}e^{\frac{-i2\pi }{3}}+\mathrm{FID}$$

(3) $$M_{\mathrm{xy}}\left(\theta =\frac{-2\pi }{3}\right)=\mathrm{STE}e^{\frac{-i2\pi }{3}}+\mathrm{STAE}e^{\frac{i2\pi }{3}}+\mathrm{FID}$$

To isolate the stimulated echo, the three acquisitions can be combined as shown in Eq. (4). Further details about the echo pathways in STEAM can be found in the Appendix (Supplementary Material).

(4) $$\mathrm{STE}=\frac{1}{3}\left(M_{\mathrm{xy}}\left(\theta =0\right)+e^{\frac{-i2\pi }{3}}M_{\mathrm{xy}}\left(\theta =\frac{2\pi }{3}\right)+e^{\frac{i2\pi }{3}}M_{\mathrm{xy}}\left(\theta =\frac{-2\pi }{3}\right)\right)$$

A chemical–shift selective fat suppression pulse was included before the third RF pulse to further suppress the fat signal arising from the free induction decay, which was not fully nulled in the phase cycling. For b > 0 s/mm^2^, data were acquired with the conventional STEAM sequence [7].

To minimize EPI readout duration, the field of view was reduced in the phase encode direction by using in-plane slice selective RF pulses as follows: the first two 90º pulses of the STEAM sequence were set along the phase-encoding direction, perpendicular to the third 90º pulse (set along the slice encoding direction), so that the echo is formed only by spins lying in the intersection of both planes 7, 30. Generalized auto-calibrating partially parallel acquisitions parallel imaging [31] with acceleration factor of 2 was used to further reduce the duration of the EPI readout. No partial Fourier sampling was used. This allowed for an EPI readout with a duration of 12.7 ms, echo train length of 24, with a matrix size of 128 × 48, an acquired resolution of 2.8 × 2.8mm^2^ reconstructed to 1.4 × 1.4 mm^2^ via zero-filling, 8 mm slice thickness, 2442 Hz/pixel bandwidth, and an echo spacing of 0.54 ms. External EPI phase correction and parallel imaging reference data were acquired at the beginning of each breath-hold. For b = 0 s/mm^2^, two repetitions of the three-point phase-cycling scheme were performed in each breath-hold (producing two b = 0 s/mm^2^ images per breath-hold), while for data acquired with b > 0 s/mm^2^, 6 diffusion encoding directions were acquired in each breath-hold, requiring 16 RR intervals per breath-hold in both cases. In total, 20 b-values were acquired: b = 0 s/mm^2^ (4 averages, with an effective SNR boost of 4√3 compared to a single image since each b = 0 s/mm^2^ image is computed from three phase-cycled images), 10 to 100 in steps of 10 s/mm^2^, 150 to 300 in steps of 50 s/mm^2^, 400 to 600 in steps of 100 s/mm^2^, 800 and 1000 s/mm^2^ (6 directions and 2 averages each), resulting in a total of 40 breath-holds required per dataset. Diffusion encoding gradients were adjusted based on the heart rate at the beginning of the STEAM-IVIM data acquisition.

### 2.2 STEAM-IVIM parameter fitting

Several fitting algorithms have been proposed in the literature to estimate the IVIM parameters [32]. The most commonly used approach assumes that since D* is typically 1 to 2 orders of magnitude larger than *D*, the perfusion component f exp(-bD*) in Eq. (1) becomes negligible at high b-values, and the signal model can be simplified to:

(5) $$S(b)/S_{0}\;\approx \;(1-f)\exp(-\mathrm{bD})$$

Using this approach, widely known as segmented fitting, the diffusion coefficient *D* can readily be obtained by fitting data acquired with *b* > *b*_*split*_ s/mm^2^ to the mono-exponential decay in Eq. (5), with *b*_*split*_ a pre-defined threshold.

For the perfusion-related parameters of the IVIM model, two fitting algorithms were considered [32]:

(1) Segmented unconstrained (SU). This algorithm finds *f* and D* by fitting all the acquired data to Eq. (1) with known *D*, using a trust region approach.
(2) Segmented constrained (SC). This algorithm obtains estimates of both *D* and *f* from the data acquired with *b* > *b*_*split*_ using Eq. (5). In the second step, only D* is estimated by fitting the acquired data to Eq. (1), with known *D* and *f.*

Simulations at varying SNR levels (5 to 100 in steps of 5 in the b = 0 data) were performed to find the optimal *b*_*split*_ and the optimal fitting algorithm. Realistic IVIM parameters in the following range were used: f∈(0−0.25), $D\in \left(0.75-1.5\right)\times 10^{-3}$ mm^2^/s, $D^{*}\in \left(10-25\right)\times 10^{-3}$ mm^2^/s, with a total of 64 combinations, and 100 datasets were generated for each SNR and IVIM parameter combination. Data were then fitted with both SU and SC algorithms, with $b_{\mathrm{split}}\in (100,200,300,400,600)$ s/mm^2^. For both fitting algorithms, box constraints as described in Eq. (6) were used 33, 34.

$4.5\times 10^{-5}\leq D\leq 1.8\times 10^{-2}\;\text{m}{\text{m}}^{2}/\text{s}$

$3.4\times 10^{-4}\leq D^{*}\leq 1.0\times 10^{-1}\;\text{m}{\text{m}}^{2}/\text{s}$

0.0005≤f≤0.9995

(6) $$D\leq D^{*}$$

Normalized root mean squared error (NRMSE) was computed at each SNR level to compare the performance of the different fitting algorithms. The optimal algorithm was then used for subsequent in vivo experiments.

### 2.3 In vivo STEAM-IVIM data processing

STEAM-IVIM data were processed using in-house developed MATLAB (MathWorks, Natick, Massachusetts, USA) software as described previously [35]. All acquired frames were visually inspected, and frames corrupted by motion were discarded. Remaining images were then aligned using a b-spline–based non-rigid registration algorithm [36], using a b = 0 s/mm^2^ stimulated echo image as a reference.

Signal intensities were then fitted to the IVIM model at every pixel using a segmented unconstrained approach as described above to obtain maps of *D*, D*, and *f* with *b*_*split*_ = 300 s/mm^2^. Prescribed b-values were corrected for the actual RR-interval on a mean over the breath-hold basis before fitting [37]. To assess myocardial microstructure, a diffusion tensor was calculated from all STEAM-IVIM data acquired with b > 150 s/mm^2^, i.e., all data acquired with b = 150, 200, 250, 300, 400, 500, 600, 800, 1000 s/mm^2^ was used for tensor calculation to ensure sufficient SNR. The diffusion tensor was calculated at every pixel using a non-linear least squares inversion, and maps of helical angle (HA), absolute value of the second eigenvector angle (|E2A|), fractional anisotropy (FA), and mean diffusivity (MD) were obtained for each subject.

Finally, to evaluate the impact of the availability of true b = 0 images in the IVIM fitting, additional fitting of the IVIM parameters excluding the b = 0 data was performed.

### 2.4 In vivo STEAM-IVIM data analysis

The left ventricle (LV) epicardial and endocardial borders were manually delineated, and median LV and interventricular septum values for the IVIM parameters (*D*, D*, and *f*) and DTCMR parameters (FA, MD, and |E2A|) were calculated after excluding the papillary muscles. Median value was chosen as summary statistic to avoid the assumption that IVIM and DTCMR parameters are normally distributed across the myocardium. Wall thickness normalized HA gradient in units of º/% was calculated from epicardium to endocardium radial profiles for quantification of HA.

To assess differences between whole LV and interventricular septum DTCMR and IVIM values, a Wilcoxon sign rank test was performed for each parameter and a P-value <0.05 was considered statistically significant.

## 3 Results

### 3.1 STEAM-IVIM simulations

**Fig. 3:**
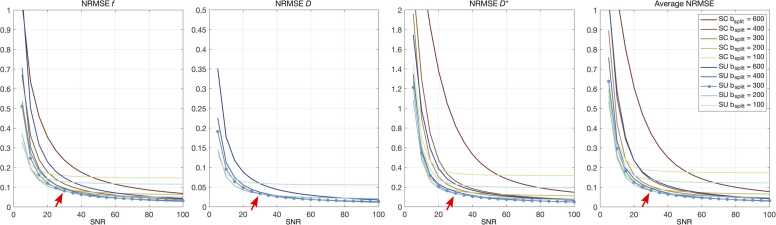
Simulation results for IVIM segmented fitting algorithms. Summary of simulation results showing the normalized root mean squared error (NRMSE) for each of the IVIM model fitted parameters (flowing blood fraction *f*, diffusion coefficient *D*, perfusion coefficient D*), and average NRMSE for all fitted parameters at different SNR levels, for both segmented unconstrained (SU) and segmented constrained (SC) fitting algorithms. Overall, the SU algorithm with b_split_ = 300 s/mm^2^ (indicated by a line with markers) outperforms the alternatives when SNR >30 (red arrow) and is close to optimal at lower b-values. *IVIM* intravoxel incoherent motion, *SNR* signal-to-noise ratio

Fig. 3 shows a summary of the simulation results for each of the IVIM parameters. A marked difference in fitting accuracy between parameters was observed, with the diffusion coefficient *D* resulting in less than 5% NRMSE for both SU and SC algorithms with b_split_ = 200, 300, and 400 s/mm^2^ for SNR > 20. Higher NMRSE was observed for the perfusion fraction *f* and perfusion coefficient D*, with errors reaching 5% only for SNR > 50 and SNR > 85, respectively, in both cases achieved when using SU fitting algorithms.

For the perfusion fraction *f*, minimal NRMSE was achieved by the SU with b_split_ = 200 s/mm^2^ for 10 < SNR < 30, while SU with b_split_ = 300 s/mm^2^ minimized error for SNR > 30, achieving an NRMSE of about 8%. A similar trend was observed for the diffusion coefficient *D* and the perfusion coefficient D*, with SU with b_split_ = 300 s/mm^2^ achieving 3% and 14% NMRSE, respectively, for data simulated with SNR = 30. Overall, the SU algorithm with b_split_ = 300 s/mm^2^ was shown to outperform the alternatives for SNR > 30 (red arrow in Fig. 3) and was used for subsequent fitting of IVIM parameters in vivo.

### 3.2 In vivo STEAM-IVIM imaging

**Fig. 4:**
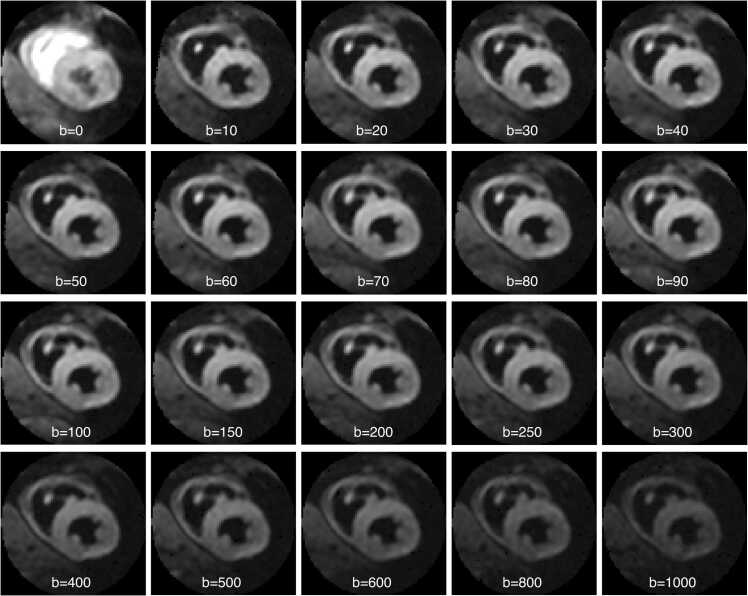
Example STEAM-IVIM images with different diffusion weighting. Example STEAM-IVIM images for a representative subject, including images acquired with all b-values from 0 to 1000 s/mm^2^, averaged over the 6 encoding directions (b > 0) and 2 repetitions (4 for b = 0). All the images are displayed with the same contrast, resulting in a saturated appearance of the blood pool in the b = 0 image. *STEAM* stimulated echo acquisition mode, *IVIM* intravoxel incoherent motion

Scans were successfully completed in all subjects, with an average scan time for the STEAM-IVIM protocol of 28 min 50 s ± 3 min 26 s. Two subjects were excluded due to errors in scan planning and one was excluded due to a history of cardiovascular disease disclosed after the scan was completed. Corrected b-values were on average 5.6% larger than prescribed b-values due to variations in heart rate during the scan (Supplementary Fig. 1). Fig. 4 shows example diffusion-weighted images for all acquired b-values averaged over the six directions and two repetitions for a representative subject. The phase-cycling approach produced good quality b = 0 s/mm^2^ images, with bright blood contrast due to the absence of spoiler gradients, and full suppression of the aliased free-induction decay signal. Good visual image quality was also observed throughout the range of acquired b-values, with a clear depiction of the left ventricular myocardium.

**Fig. 5:**
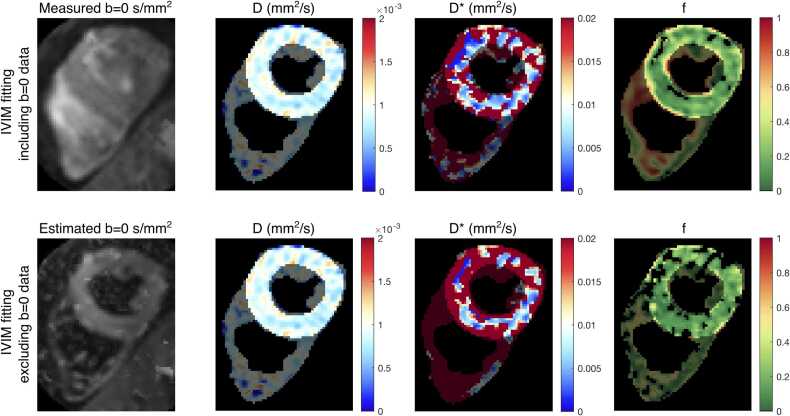
Effect of true b = 0 image in IVIM fitting. Example IVIM maps, including diffusion coefficient *D*, perfusion coefficient D*, and flowing blood fraction *f*, for data fitting including (top row) and excluding (bottom row) true b = 0 s/mm^2^ images. Excluding b = 0 images results in unstable fitting and patchy *f* maps, with large areas of the myocardium fitted to *f* ∼ 0. *IVIM* intravoxel incoherent motion

Example IVIM maps fitted from all acquired data and fitted from a subset of data excluding b = 0 s/mm^2^ images are shown in Fig. 5 for a representative subject. When excluding the true b = 0 image, fitting of the *f* and D* parameter becomes unstable, resulting in large areas of the myocardium with *f* ∼ 0.

**Fig. 6:**
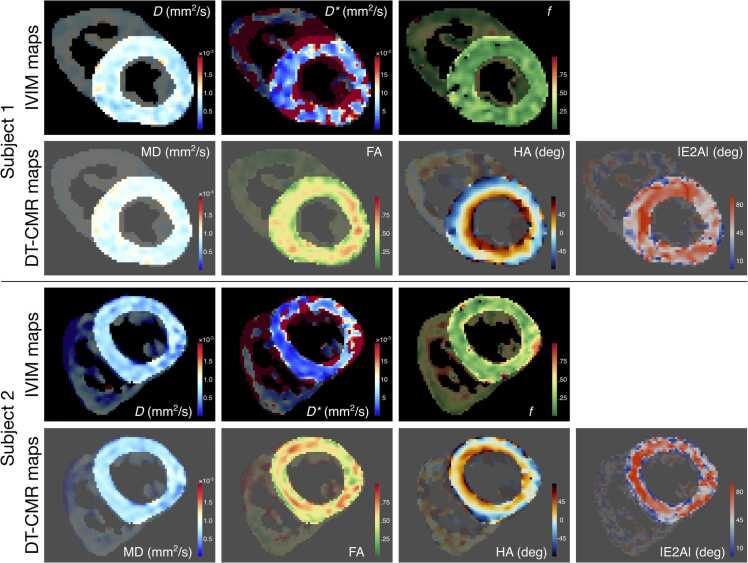
Example IVIM and DTCMR maps. Example IVIM maps (top row), including diffusion coefficient *D*, perfusion coefficient D*, and flowing blood fraction *f*, and DTCMR maps (bottom row), including mean diffusivity (MD), fractional anisotropy (FA), helix angle (HA), and absolute E2A, for two representative subjects. *DTCMR* diffusion tensor cardiovascular magnetic resonance, *IVIM* intravoxel incoherent motion

**Fig. 7:**
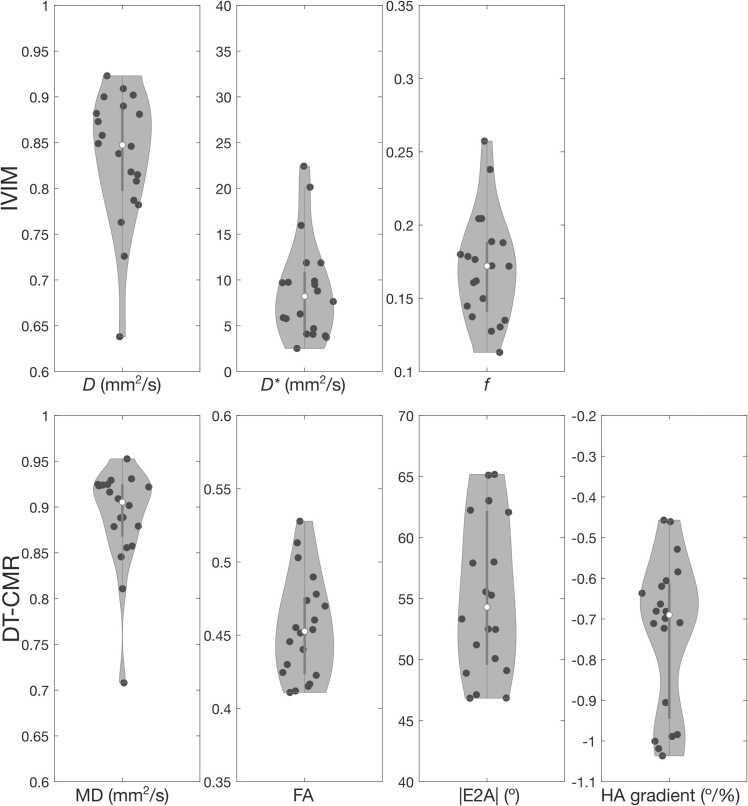
Cohort results for IVIM and DTCMR parameters. Summary of cohort results showing violin plots for IVIM (top row) and DTCMR (bottom row) parameters. Dots in each plot represent the median value across the LV myocardium for each parameter for each subject. *DTCMR* diffusion tensor cardiac magnetic resonance, *IVIM* intravoxel incoherent motion, *LV* left ventricle, *MD* mean diffusivity, *FA* fractional anisotropy, *HA* helix angle

Example IVIM maps and DTCMR maps, including MD, FA, HA, and absolute E2A, for two representative subjects are shown in Fig. 6. A summary of cohort results is presented in Fig. 7, including IVIM and DTCMR parameters. Mean LV IVIM parameter estimates across the 20 healthy subjects were: *D* = 0.83 ± 0.07 × 10^−3^ mm^2^/s, D* = 8.92 ± 5.44 × 10^−3^ mm^2^/s, and *f* = 17.09 ± 3.66%, while mean DTCMR parameters included MD = 0.88 ± 0.05 × 10^−3^ mm^2^/s, FA = 0.45 ± 0.03, absolute E2 angle = 55.29 ± 6.38º, helix angle gradient = −0.73 ± 0.18º/%, which are all within range of previously reported DTCMR parameters in healthy subjects [38]. Septal IVIM parameters were not significantly different to whole LV values, with *D* = 0.84 ± 0.07 × 10^−3^ mm^2^/s (P = 0.06), D* = 10.61 ± 8.38 × 10^−3^ mm^2^/s (P = 0.11), and *f* = 16.20 ± 3.80% (P = 0.06). Septal DTCMR parameters, including MD (0.90 ± 0.06 × 10^−3^ mm^2^/s, P = 0.01), absolute E2 angle (60.09 ± 9.76º, P = 0.002), and helix angle gradient (−0.79 ± 0.21º/%, P = 0.02) were significantly higher than LV values, whereas septal FA (0.44 ± 0.03, P = 0.052) was not significantly different to the LV metric. Violin plots showing detailed septal IVIM and DTCMR parameters are shown in Supplemental Fig. 2.

## 4 Discussion

This is the first study to demonstrate the feasibility of cardiac IVIM imaging in the human heart in vivo using a STEAM sequence and produce normal values for future reference in clinical studies. To enable the acquisition of true b = 0 s/mm^2^ images, we introduced a phase-cycling approach and evaluated the proposed method in 20 healthy subjects. Preliminary results demonstrate the feasibility of our method for the simultaneous assessment of cardiac diffusion tensor parameters, and microvascular perfusion through the perfusion coefficient and perfusion fraction.

The ability of the IVIM approach to provide information about microvascular perfusion without the need for exogenous contrast agents has motivated interest in this technique for cardiac imaging over the last two decades. While pioneering work using a STEAM sequence for cardiac IVIM was reported in a canine study with external cardiac pacing [27], much of the published research on cardiac IVIM in humans has used monopolar SE sequences designed for imaging static anatomy such as the brain 15, 34, 35, 36, 37. Such techniques are known to produce inconsistent image quality in the heart, where complex physiological cardiac and respiratory motion can lead to erroneous estimation of diffusion and perfusion parameters [15]. To address this issue, cardiac IVIM with motion–compensated SE sequences has been explored in the literature (Supplementary Table 1 for details) 16, 17, 34. While the use of motion-compensated gradients has been successful in SE DTCMR [23] for minimizing the effect of physiological motion, recent simulation and ex-vivo studies have shown that such approaches make the received signal relatively insensitive to changes in perfusion 24, 25. Indeed, Alemany et al. performed a realistic simulation study [25] comparing STEAM, monopolar SE, and motion–compensated SE sequences, showing that while both monopolar SE and STEAM are sensitive to changes in perfusion, the magnitude of signal decay using monopolar SE is significantly smaller than when using STEAM. This may result from the longer diffusion times of STEAM, which allow water molecules to perfuse and disperse large distances, increasing the sensitivity of the signal to changes in perfusion. Similar results were found in [24] where both in simulation and *ex-vivo* experiments, monopolar SE sequences exhibited a low sensitivity to changes in perfusion, which decreased further when motion‐compensated diffusion-encoding gradients were used. As demonstrated here, breath-hold ECG-triggered STEAM-based IVIM offers robustness against physiological motion and the ability to detect changes in myocardial perfusion. However, despite being a promising approach, before our study, STEAM-IVIM had not been applied to the human heart in vivo.

In this work, we extended our previously introduced cardiac STEAM sequence 7, 30 to enable the acquisition of b-values from 0 to 1000 s/mm^2^. We introduced a fat-suppressed three-point phase-cycling approach for the acquisition of true b = 0 s/mm^2^ images, which removed the need for spoiler gradients, and produced good quality images with the robust and effective fat suppression typical of STEAM DTCMR. Such images enabled reliable fitting of IVIM parameters, with maps of the diffusion coefficient *D* and the perfusion fraction *f* with values generally consistent across the myocardium, as expected for healthy subjects. D* maps were visually less homogeneous, likely due to higher errors in the fitting process as demonstrated in our prior simulation study where the fitting of D* resulted in a higher NRMSE at all SNR levels (Fig. 3).

Differences in signal homogeneity were observed when comparing the interventricular septal region and the free wall. This can be observed in the b = 0 phase-cycled images, in the diffusion-weighted images especially at higher b-values, and in the final parametric maps. This is a known feature of DTCMR, and indeed of several CMR techniques including T1, T2, and T2* mapping, which are sensitive to magnetic field inhomogeneities present in the lung/heart interface near the myocardial free wall.

The average estimated *D* value across subjects was 0.83 ± 0.07 × 10^−3^ mm^2^/s in our study, which slightly lower than published literature in STEAM-IVIM. On the other hand, the average D* (8.92 ± 5.44 × 10^−3^ mm^2^/s) and *f* (17.09 ± 3.66%) in this study were higher than previous STEAM-IVIM studies. However, a direct comparison of the estimated IVIM values to previous literature is not possible since previous studies were preclinical canine in vivo studies acquired at end-diastole over a narrower range of b-values [27] or perfused heart ex-vivo [24] experiments.

Previously introduced approaches for DTCMR based on STEAM use 2 b-values and several averages: typically, 2 averages for a reference b-value of around 150 s/mm^2^, and 8 or more averages of a higher b-value of ∼600 s/mm^2^, for a total of around 10 breath-holds per slice 10, 30, 35, 39. To obtain DTCMR parameters in this study, we used all b-values from 150 to 1000 s/mm^2^, each of them acquired with 2 averages, resulting in 18 breath-holds in total. Despite differences in the acquisition protocol, average DTCMR parameters were comparable to prior studies [38], which reported MD = 0.96 ± 0.05 × 10^−3^ mm^2^/s, FA = 0.44 ± 0.03, absolute E2A = 61.8 ± 4.8º, HA gradient = −0.74 ± 0.09º/% for STEAM acquisitions in systole in healthy subjects, compared to MD = 0.88 ± 0.05 × 10^−3^ mm^2^/s, FA = 0.45 ± 0.03, absolute E2A = 55.29 ± 6.38º, HA gradient = −0.73 ± 0.18º/% in this study.

It is worth noting that both the IVIM diffusion coefficient *D* and the DTCMR parameter MD are measuring the same apparent coefficient of water diffusivity. Differences in these estimates might be explained because MD was derived from a tensor model which takes directionality of the diffusivity into account, while a scalar model was used for the IVIM parameters. Previous studies [24] have shown that using a tensor model for the *D* and D* parameters in IVIM can reduce bias, particularly in low SNR and high diffusion anisotropy scenarios. A comprehensive comparison of tensor versus scalar fitting of the IVIM parameters is out of the scope of this study and remains future work.

The IVIM perfusion parameters *f* and D* are related to conventional quantitative CMR perfusion parameters by the density and size of vessels in the capillary network. Indeed, the IVIM perfusion fraction is related to the myocardial blood volume (MBV) by *f* *=* MBV*/f*_w_ [40], with *f*_w_ the water content fraction detected by MRI. On the other hand, the IVIM perfusion coefficient D* is related to the mean capillary length 〈*l*〉 and mean velocity 〈*v*〉 by D* = 〈*l*〉〈*v*〉/6, and therefore depends on the number of capillary branches traversed. Using these relations, we can also relate the myocardial blood flow (MBF) to the IVIM parameters by MBF = (6 *f*_w_/*L*〈*l*〉) fD*, where *L* is the total capillary length.

Simultaneous contrast-free assessment of myocardial perfusion by IVIM and microstructure by DTCMR could provide a step-change in clinical CMR, potentially enabling contrast-free assessment of most patient populations. It might also provide additional insights into cardiac conditions such as hypertrophic cardiomyopathy, where microvascular dysfunction has been associated with ventricular dysfunction and heart failure [41] and microstructural dynamics have been shown to be impaired [8]. Similarly, our approach could provide a contrast-free alternative for the assessment of myocardial perfusion in patients where the use of gadolinium-based contrast agents might be contraindicated due to impaired renal function, such as in diabetic and ureic cardiomyopathy.

## 5 Limitations

The aim of this work was to demonstrate the feasibility of STEAM-IVIM in vivo and provide a reliable estimation of IVIM parameters that might be used as reference values for healthy myocardium. For this, we used 20 b-values ranging from 0 to 1000 s/mm^2^. However, this resulted in long scan times of about 30 min per slice, hindering the clinical translation of our technique at present. Comparable studies have used between 9 and 13 b-values (Supplementary Table 1 for details), and future work includes optimization of the number of b-values required for our STEAM-based approach to produce reliable estimates of the IVIM parameters and the application of denoising techniques to reduce the number of required data points [42]. Our initial demonstration of the technique was performed in healthy volunteers without the pharmacological-induced stress that is typical for first-pass CMR perfusion studies, but future studies will investigate the potential of this technique to assess ischemia in myocardial pathologies and the possibility of using abbreviated STEAM-IVIM protocols during stress.

## 6 Conclusions

We have demonstrated for the first time the feasibility of performing cardiac IVIM in vivo in the human heart with a phase-cycled STEAM sequence which provides good quality true b = 0 images for a reliable fitting of the IVIM parameters. This approach shows potential for the simultaneous assessment of microvascular perfusion and myocardial microstructure from a single scan, and without the need for external contrast agents, that may provide new insights, earlier diagnosis and precision prognosis in cardiac pathology at microscopic scales.

## Funding

This work was funded by the 10.13039/501100000266Engineering and Physical Sciences Research Council, grant EP/X014010/1, and 10.13039/501100000274British Heart Foundation grant RG/19/1/34160 and RG/F/23/110115. This project has been made possible in part by grant 2024-337787 from the Chan Zuckerberg Initiative DAF, an advised fund of the 10.13039/100000923Silicon Valley Community Foundation.

## Author contributions

Camila Munoz: Writing—review and editing, Writing—original draft, Visualization, Software, Methodology, Investigation, Formal analysis, Data curation, Conceptualization. Pedro F. Ferreira: Writing—review and editing, Software, Methodology, Formal analysis. Eunji Lim: Writing—review and editing, Methodology. Sonia Nielles-Vallespin: Writing—review and editing, Supervision, Resources, Funding acquisition, Formal analysis, Conceptualization. Dudley J. Pennell: Writing—review and editing, Resources, Funding acquisition. Andrew D. Scott: Writing—review and editing, Supervision, Methodology, Funding acquisition, Formal analysis, Conceptualization.

## Declaration of competing interests

The authors declare the following financial interests/personal relationships which may be considered as potential competing interests: The Cardiovascular Magnetic Resonance Unit at the Royal Brompton Hospital receives research support from Siemens.
