# Supplementary material for: Simultaneous non-contrast assessment of cardiac microstructure and perfusion in vivo in the human heart
Source: J Cardiovasc Magn Reson. 2024 Nov 30;27(1):101129. doi: 10.1016/j.jocmr.2024.101129 (PMC12182817; doi:10.1016/j.jocmr.2024.101129)
Supplement: Supplementary Fig. 1 — Supplementary material. [file mmc1.docx]

**Appendix - Phase Cycling Scheme**


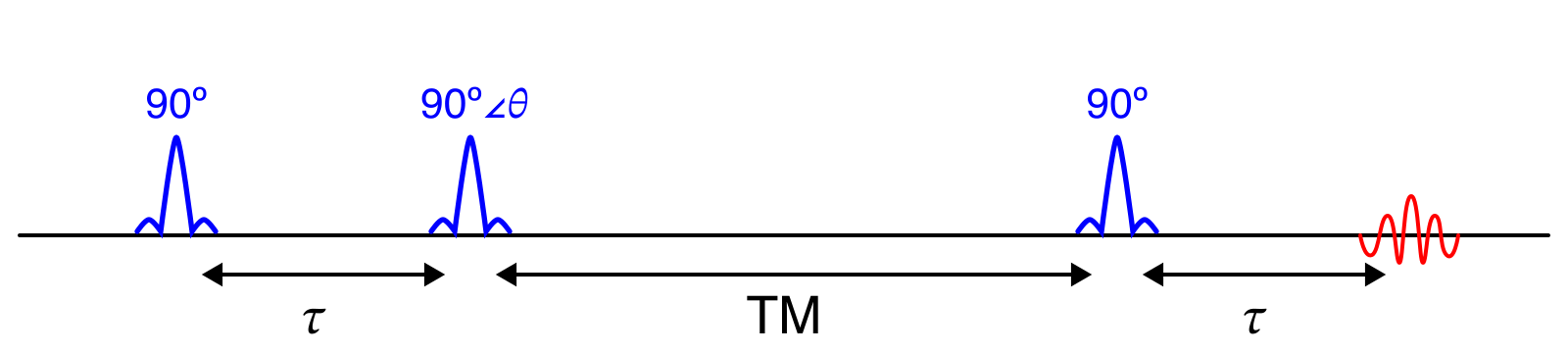


In absence of diffusion encoding gradients, the three 90º RF pulses of the STEAM sequence result in three coherent echo pathways: the desired stimulated echo signal, a stimulated anti-echo signal, and a free induction decay signal arising from the third 90º RF pulse.

If we consider a longitudinal magnetization M prior to the application of the first 90º RF pulse, in the time 𝜏 between the first and second RF pulses, $M_{xy}=M$ decays with T2* and accumulates a phase 𝜙_1_ depending on off resonance and field inhomogeneities ($\Delta\omega_{OFF}$) at location r_1_, so that just before the application of the second 90º RF pulse the signal for the voxel at r_1_ is:

$$M_{xy}=M f\left( \tau,T2^{*} \right)e^{-i\phi_{1}}$$

with $\phi_{1}=\Delta\omega_{OFF}\left( r_{1} \right)\tau$ and where $f\left( \tau,T2^{*} \right)$ describes the reduction in signal magnitude due to intravoxel dephasing.

After the second 90º RF pulse is applied with phase 𝜃, part of the magnetization returns to the longitudinal axis and recovers back to its initial state over the mixing time TM with T1. With TM≫T2*, any residual transverse magnetization will decay rapidly. Right before the third 90º RF the longitudinal magnetization can be therefore expressed as:

$$M_{z}=\frac{1}{2}M f\left( \tau,T2^{*} \right)\left( e^{i\left( \phi_{1}-\theta\right)}+e^{-i\left( \phi_{1}-\theta\right)} \right)e^{-\frac{TM}{T1}}+\left( 1-e^{-\frac{TM}{T1}} \right)M_{0}$$

Finally, the third 90º RF pulse tips this magnetization into the transverse plane

$$M_{xy}=\frac{1}{2}M f\left( 2\tau,T2 \right)e^{i\left( \phi_{2}-\phi_{1}+\theta\right)}e^{-\frac{TM}{T1}}$$

$$+\frac{1}{2}Mf\left( 2\tau,T2^{*} \right)e^{i\left( \phi_{2}+\phi_{1}-\theta\right)}e^{-\frac{TM}{T1}}$$

$$+M_{0}f\left( \tau,T2^{*} \right)\left( 1-e^{-\frac{TM}{T1}} \right)e^{-i\phi_{2}}$$

with $\phi_{2}=\Delta\omega_{OFF}\left( r_{2} \right)\tau$. If the imaged object is at the same spatial location (i.e. at the same cardiac phase in two consecutive heart beats), then $r_{1}=r_{2}$ and $\phi_{1}=\phi_{2}=\phi$, and the previous expression can be simplified to

$$M_{xy}=\frac{1}{2}M f\left( 2\tau,T2 \right)e^{-\frac{TM}{T1}}e^{i\theta}$$

$$+\frac{1}{2}Mf\left( 2\tau,T2^{*} \right)e^{i2\phi}e^{-\frac{TM}{T1}}e^{-i\theta}$$

$$+M_{0}f\left( \tau,T2^{*} \right)\left( 1-e^{-\frac{TM}{T1}} \right)e^{-i\phi}$$

Where the first component $STE=\frac{1}{2}M f\left( 2\tau,T2 \right)e^{-\frac{TM}{T1}}$ corresponds to the desired stimulated echo; the second component $STAE= \frac{1}{2}Mf\left( 2\tau,T2^{*} \right)e^{i2\phi}e^{-\frac{TM}{T1}}$ is a T2* and off-resonance affected stimulated anti-echo; and ${FID=M}_{0}f\left( \tau,T2^{*} \right)\left( 1-e^{-\frac{TM}{T1}} \right)e^{-i\phi}$ corresponds to the high-signal free induction decay from the third RF pulse.

The signal can therefore be written as

$$M_{xy}\left( \theta\right)=STEe^{i\theta}+STAEe^{-i\theta} +FID$$

To isolate the stimulated echo, a minimum of three acquisitions with different phases 𝜃 is required. Three-point phase cycling methods use $\theta=0, \frac{2\pi}{3}, -\frac{2\pi}{3}$ so that in three consecutive acquisitions the following signal is acquired

$$M_{xy}\left( \theta=0 \right)=STE+STAE+FID$$

$$M_{xy}\left( \theta=\frac{2\pi}{3} \right)=STEe^{\frac{i2\pi}{3}}+STAEe^{\frac{-i2\pi}{3}}+FID$$

$$M_{xy}\left( \theta=\frac{-2\pi}{3} \right)=STEe^{\frac{-i2\pi}{3}}+STAEe^{\frac{i2\pi}{3}}+FID$$

The complex signal from the three acquisitions can therefore be combined to obtain the isolated STE signal by:

$$STE=\frac{1}{3}\left( M_{xy}\left( \theta=0 \right)+e^{\frac{-i2\pi}{3}}M_{xy}\left( \theta=\frac{2\pi}{3} \right)+e^{\frac{i2\pi}{3}}M_{xy}\left( \theta=\frac{-2\pi}{3} \right) \right)$$

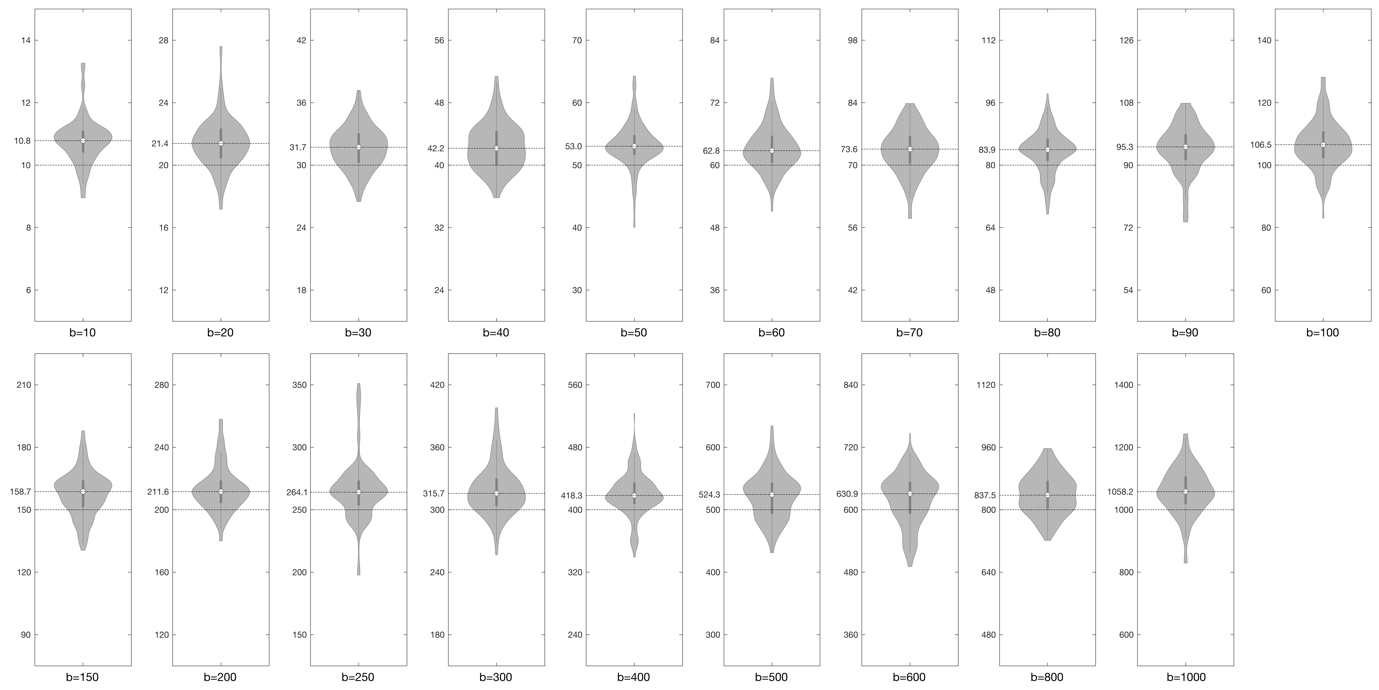


Supplemental Figure 1. Violin plots of prescribed vs actual corrected b-values for all subjects scanned in this study. Median corrected b-value is indicated in each plot. Corrected b-values were on average 5.6% larger than prescribed b-values due to variations in heart rate.


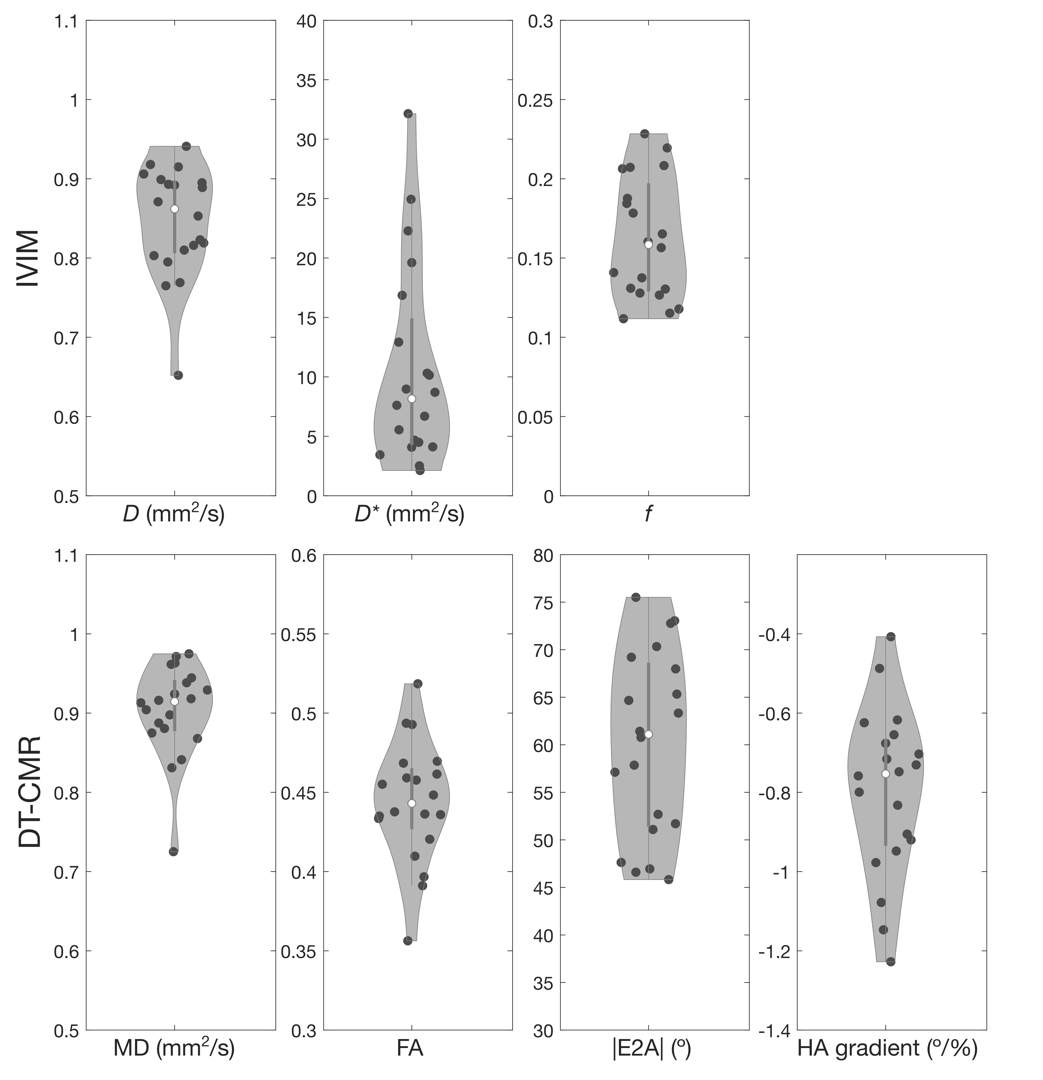


Supplemental Figure 2. Summary of cohort results showing violin plots for septal IVIM (top row) and DTCMR (bottom row) parameters. Dots in each plot represent the median value across septal segments for each parameter for each subject.

|  | Subjects (n) | Sequence; Field strength |  | D (10^-3^ mm^2^/s) | f (%) | D* (10^-3^ mm^2^/s) | Range of b-values (n) |
| --- | --- | --- | --- | --- | --- | --- | --- |
| Callot et al 2003 | Canine (10) | **STEAM**; 1.5T | **Baseline** | **1.26 ± 0.10** | **8.24 ± 1.27** | **12.87 ± 2.56** | 0 to 600 (14) |
|  |  |  | Adenosine | 1.25 ± 0.08 | 10.31 ± 1.54 | 14.79 ± 2.27 |  |
|  |  |  | Post-mortem | 0.90 ± 0.04 | 0.86 ± 0.65 | - |  |
| Delattre et al 2012 | Healthy subjects (12) | Bipolar SE; 1.5T |  | 2.43 ± 0.98 | 15.0 ± 4.6 | 76.3 | 0 to 550 (13) |
| Froeling et al 2014 | Healthy subjects (1) | Bipolar SE; 3T |  | 1.67 ± 0.49 | 27.0 ± 16.0 | 52.68 ± 52.61 | 0 to 300 (6) |
| von Deuster et al 2015 | Swine myocardial infarction (2) | M2-SE; 1.5T | Baseline | 1.40 ± 0.20 | ~15.0 to 25.0 | ~15.0 | 0 to 1000 (16) |
|  |  |  | Post-mortem | 1.20 ± 0.30 | 5.0 ± 16.0 | - |  |
| Abdullah et al 2016 | Perfused guinea pig hearts (7) | Monopolar SE; 7T | Baseline  Low perfusion | ~0.50 to 0.75  ~0.50 to 0.75 | ~15.0  ~10.0 | ~20  ~10 | 1 to 1020 (13) |
| Moulin et al 2016 | Healthy subjects (10) | Monopolar SE; 1.5T |  | 1.41 ± 0.09 | 12.2 ± 1.3 | 43.6 ± 9.2 | 5 to 400 (9) |
| Spinner et al 2017 | Healthy subjects (8) | M2-SE; 1.5T | LSQ  BSP | 1.63 ± 0.28  1.51 ± 0.14 | 13.13 ± 19.81  13.11 ± 5.95 | 201.45 ± 313.23  13.11 ± 14.53 | 20 to 300 (11) |
| An et al 2018 | Healthy subjects (12) + MI patients (20) | Monopolar SE; 3T | Healthy  24h post MI  3d post MI  7d post MI  30d post MI | 1.53 ± 0.21  1.41 ± 0.10  1.30 ± 0.10  1.37 ± 0.10  1.39 ± 0.12 | 15.31 ± 3.0  12.03 ± 2.0  9.89 ± 2.0  11.09 ± 2.0  12.13 ± 2.0 | 109.33 ± 15.73  60.09 ± 9.17  59.03 ± 10.03  61.20 ± 10.19  62.29 ± 9.03 | 0 to 800 (9) |
| Spinner et al 2019 | Perfused swine heart (1) | Monopolar SE, Bipolar SE, M2-SE, and **STEAM**; 1.5T | Monopolar SE  Bipolar SE  M2-SE  **STEAM** | 1.24 ± 0.13  1.27 ± 0.11  1.36 ± 0.15  **0.87 ± 0.14** | 10.78 ± 1.85  10.97 ± 3.91  11.81 ± 2.15  **8.12 ± 3.73** | 5.60 ± 0.55  5.00 ± 1.55  5.77 ± 0.67  **4.45 ± 1.60** | 10 to 1000 (12) |
| Wu et al 2020 | HCM patients (55) | Monopolar SE; 3T | LGE+  LGE- | 1.89 ± 0.14  1.63 ± 0.12 | 10.8 ± 1.29  12.5 ± 1.26 | 34.9 ± 6.6  55.2 ± 11.4 | 0 to 800 (10) |
| Zhang et al 2020 | Healthy subjects (10) | Monopolar SE; 3T | Data b>=10  All data | 1.83 ± 0.27  1.83 ± 0.27 | 15 ± 2  26 ± 4 | 44.01 ± 6.26  138.02 ± 13.08 | 0 to 400 (10) |
| Zhang et al 2022 | Healthy subjects (10) | Monopolar SE; 3T | Test  Re-test | 1.73 ± 0.09  1.74 ± 0.0.8 | 21 ± 4  22 ± 4 | 110.57 ± 21.70  107.70 ± 15.70 | 0 to 400 (9) |

Supplemental Table 1. Review of literature published on cardiac IVIM, excluding publications with non-physical diffusivity values reported (see Moulin K et al. JMRI 2023:58(6), 1990–91). STEAM-IVIM data is highlighted in bold
